# Application of Genomic Random Regression Models for Genetic Parameter Estimations of Female Fertility Traits in Different Parities in German Holsteins

**DOI:** 10.1111/jbg.70027

**Published:** 2025-11-14

**Authors:** Sina Sakhaei‐far, Tong Yin, Sven König

**Affiliations:** ^1^ Institute of Animal Breeding and Genetics Justus‐Liebig‐University Gießen Gießen Germany; ^2^ Zhejiang Key Laboratory of Dairy Cattle Genetic Improvement and Milk Quality Research Wenzhou P. R. China

**Keywords:** female fertility, genetic parameters, genomic random regressions

## Abstract

The aim of the present study was to infer genetic (co) variance components and to estimate parity‐specific breeding values for the female fertility traits non‐return rate after 56 days, the interval from calving to first service and days open by applying random regression models on a time‐dependent parity scale. In this regard, we considered a female fertility dataset comprising 592,829 records on 190,269 German Holstein cows and heifers kept in 45 large‐scale dairy contract herds. From a subset of 21,316 cattle with phenotypic records, (imputed) 50 K genotypes were available. The applied genomic random regression model considered Legendre polynomials of order 2 for the additive‐genetic effects along the parity scale, and combined pedigree and genomic relationships through the **H**‐matrix. Results were compared with genetic parameter estimates from a multiple‐trait model, considering the same fertility trait in different parities as different traits. From both modelling approaches, we observed the trend of increasing genetic variances and heritabilities with increasing parity. Especially for the non‐return rate, the genetic variance in heifers was substantially smaller than in all parities of cows. With regard to the random regression model, genetic correlations between the same fertility traits from adjacent parities were close to 1, but gradually declined with increasing parity distances. Small genetic correlations were also estimated between non‐return rates in heifers with non‐return rates in all cow parities, i.e., 0.50 with parity 1, 0.44 with parity 2, 0.41 with parity 3, 0.35 with parity 4, 0.33 with parity 5, and 0.25 with parity 6. A similar pattern for genetic correlations in the same traits across parities was confirmed from the multiple‐trait model application. Estimated breeding values for all fertility traits in different parities of sires with at least 10 phenotyped daughters per trait (estimates from the random regression model) were correlated with their official breeding indexes from the national genetic evaluation. In this regard, moderate differences were observed when comparing breeding value correlations for non‐return rates in heifers with respective correlations in all cow parities. From a practical breeding perspective, the most important results were the rather small genetic correlations for the same traits in different parities (e.g., 0.24 between calving to first service in parities 1 and 6), and differing breeding value correlations with other breeding indexes in different parities. These findings suggest the implementation of specific genetic evaluations for specific cow parities, as an extension to the existing separation between heifer and cow fertility traits. Parity‐specific breeding value correlations from the random regression and the multiple‐trait model considering the sires with at least 10 daughters were larger than 0.85, suggesting only minor re‐rankings of sires from the two different modeling approaches.

## 1 Introduction

As outlined by, e.g., Gernand and König (2017), female fertility in dairy cattle can be defined as the ability to show heat or maturity, to conceive, and to recycle. In consequence, female fertility is a very complex trait, and accordingly, new breeding indexes have been developed to cover both major fertility components, i.e., the start of a new cycle after calving and the success of an insemination (e.g., Gonzalez‐Recio and Alenda 2005; Jorjani 2006). Furthermore, in most countries, multiple‐trait models (MTM) have been developed for female fertility genetic evaluations, aiming at a separation of heifer and cow fertility traits (e.g., Muuttoranta et al. 2019). Nevertheless, in spite of all these efforts, the fertility status of Holstein Friesian cows gradually declined or stagnated in many countries during the past decades, even in the genomics era (Veerkamp et al. 2015). Efficient breeding approaches for improved female fertility are hampered due to the strong environmental component. In consequence, also the application of the so‐called ‘genomic herd management’, i.e., utilisation of early genomic breeding values to predict later phenotypes, yielded limited success for low heritability conception rates with a correlation coefficient between breeding values and phenotypes of −0.05 (Strabel 2024).

In addition to the strong environmental effect, the complexity of female fertility genetic mechanisms (Gajbive et al. 2018) might be a major challenge to develop accurate genomic predictions. The complexity of physiological mechanisms implies the activity of different genes in reproductive processes with progressive time and in different environments, as outlined by Beerda et al. (2008). Alterations of genetic mechanisms with progressing time or environmental alterations indicate that different genes are ‘switched on or off’ in the course of lactation as well as across lactations (König and May 2018). From a quantitative‐genetic perspective, random regression models (RRM) have the greatest potential for depicting such underlying genomic particularities. For test‐day traits, RRM are implemented in national genetic evaluations for more than two decades for the estimation of breeding values and genetic parameters by days in milk (e.g., Swalve 2000). RRM have the potential for a better correction of environmental effects than multiple‐trait (MTM) or repeatability models in case of dense longitudinal observations (Swalve 1995). Khanal et al. (2022) outlined the advantages of RRM for feed efficiency traits, due to the pronounced environmental alterations within short periods and respective effects on variance component estimates. The superiority of RRM over, example, MTM, is unclear for traits with repeated measurements at greater distances. In this regard, as an extension for most of the female fertility traits with only one single observation per lactation or for survival analysis, Schaeffer (2004) suggested modeling random regressions by parity. An RRM approach considering genetic parameter alterations in different parities was presented by Veerkamp et al. (1999) for cow longevity. Accordingly, Oliveira et al. (2020) outlined the advantages of RRM in case of a censored data structure and a substantial trait record reduction with increasing aging. In consequence, they applied RRM for genetic analyses of survival traits in beef cattle. In analogy, also for female fertility traits observed at great distances, effects of selection and cullings imply a quite large number of records in heifers and in first parity cows, but an obvious data decline in later lactations.

The above‐mentioned RRM applications for survival or longevity during the animals' lifespan considered pedigree relationship matrices. For within‐animal observations being far apart, strong fluctuations of genetic covariances in the same traits along the time trajectory are expected, due to evolutionary effects including selection, migration and drift (Do and Whitlock 2023). RRM has greater flexibility in modeling appropriate covariance functions, considering the particularities of the trait biology (Kirkpatrick et al. 1990). Additional value in the genomic era in this regard might be due to the availability of dense SNP markers, capturing genetic covariances more accurately than pedigree‐based approaches (Beaulieu et al. 2014).

Consequently, the aim of the present study was to utilise comprehensive phenotype datasets from a large number of genotyped Holstein cows kept in large‐scale German contract herds to evaluate genomic RRM for the female fertility traits non‐return rate after 56 days (NRR56), interval from calving to first service (CTFS) and days open (DO). Variance components, heritabilities and genetic correlations in the same traits from different parities were compared with respective results from MTM. The estimated breeding values for NRR56, CTFS and DO in different parities from sires with daughter records were correlated with their official breeding values for all indexes included in the German overall net merit index.

## 2 Materials and Methods

### 2.1 Cow Traits, Pedigree and Genotypes

The female fertility dataset comprised 592,829 records of 190,269 Holstein cows and heifers kept in 45 large‐scale dairy contract herds from the German federal states of Hesse, Mecklenburg‐West Pomerania and Berlin‐Brandenburg. These herds represent the original nucleus used for implementing genomic selection in Germany based on cow training sets (e.g., Klein et al. 2021). Female fertility traits for CTFS, DO and NNR56 were from the years 2010 to 2022. The trait data structure in parities 0 (heifer records for NNR56) to 6 is given in Table 1.

**TABLE 1:** Number of observations and descriptive statistics for the female fertility traits non‐return rate after 56 days (NRR56), interval from calving to first service (CTFS) and days open (DO) by parity (parity 0 = heifers).

Trait	Parity	No. of observations	Mean	SD
NRR56	0	166,736	0.71	0.45
CTFS	0	0	—	—
DO	0	0	—	—
NRR56	1	154,352	0.53	0.49
CTFS	1	158,132	77.09	27.40
DO	1	128,808	117.43	58.75
NRR56	2	114,446	0.49	0.50
CTFS	2	117,442	77.57	26.86
DO	2	89,513	123.00	60.15
NRR56	3	146,790	0.47	0.49
CTFS	3	150,519	79.97	27.15
DO	3	102,042	127.13	60.21
NRR56	4	41,938	0.46	0.49
CTFS	4	43,076	80.45	27.33
DO	4	28,808	128.67	60.73
NRR56	5	20,958	0.47	0.49
CTFS	5	21,512	81.23	27.28
DO	5	13,346	128.94	59.76
NRR56	6	9127	0.46	0.49
CTFS	6	9380	81.22	26.93
DO	6	5503	128.76	59.37

The pedigree of the 190,269 female cattle with phenotypes considered at least three previous generations (sire and dam, respective grandparents and respective great‐grandparents). Oldest ancestors were born in 1920. The 190,269 cattle with phenotypes had 5787 different sires, 7116 different maternal grand‐sires and 2850 different paternal grand‐sires.

In a subset, 21,316 cattle with phenotypic records were genotyped. Of these, 5403 animals were genotyped with the *Illumina Bovine SNP50 v2 Bead Chip*, and 15,913 animals were genotyped with the *Illumina Bovine Eurogenomics 10 K low‐density chip*. Among the pool of sires and grand‐sires, a further 948 males were genotyped with the *Illumina Bovine SNP50 v2 Bead Chip*. Cattle with low‐density 10 K genotypes were imputed to the 50 k panel by the project partner vit Verden using their algorithm as applied for routine national genetic evaluations (Segelke et al. 2012). Genotype quality control was carried out using the software package PLINK (Purcell et al. 2007). The applied filters encompassed the following criteria: a minor allele frequency of 0.05 (exclusion of 3.677 SNP), a minimum call rate of 0.9 (exclusion of 866 SNP) and significant deviation (*p*‐value < 1 × 10^−6^) from Hardy–Weinberg equilibrium (exclusion of 4 SNP). Genomic relationships among all genotyped animals were smaller than 0.95. For one genotyped heifer, we identified Mendelian conflicts, i.e., inconsistencies when comparing the individual genotype with the parental genotypes. The genotype for this animal was ignored. Finally, after filtering, 41,129 SNP from 22,633 genotyped animals were available for the ongoing genomic studies.

### 2.2 Statistical Models

The applied MTM simultaneously considered the same trait from different parities as different traits. Hence, the MTM for NRR56 included 7 traits (heifers and 6 cow parities), and for CTFS and DO 6 traits (the 6 cow parities).

The general statistical model defined in matrix notation for the multiple‐trait analyses was:

$$y=\mathrm{Xb}+\mathrm{Zu}+\left(\mathrm{SSs}\right)+e$$

where **y** was the vector for the same fertility trait in different lactations; **b** was the vector for fixed effects including the combined effect of herd‐year‐season of insemination and age of cow at insemination (in months), **u** was the vector for additive‐genetic effects with **u** ~ *N*(**0**, **H** σ^2^_a_), and σ^2^_a_ denoting the additive genetic variance and **H** denoting the combined (pedigree and genomics) relationship matrix constructed according to Legarra et al. (2009); **s** was the vector for random service sire effects on DO and NRR56 with **s** ~ *N*(**0**, **I** σ^2^_s_), and σ^2^_s_ denoting the service sire variance and **I** denoting an identity matrix for the 6580 service sires, and **e** was the vector for the residual effects with **e** ~ *N*(**0**, **I** σ^2^_e_), and σ^2^_e_ denoting the residual variance and **I** denoting an identity matrix for the cows and heifers with observations. **X**, **Z** and **SS** were incidence matrices for **b**, **u** and **s**, respectively.

The variance–covariance structure for random effects between the same fertility traits in different parities *i* and *j* with random service sire effects (NRR56, DO; CTFS without random service sire effects) was:

$$\mathrm{var}\left[\begin{array}{c} \begin{array}{c} u_{i}\\ u_{j}\\ s_{i}\\ s_{j} \end{array}\\ e_{i}\\ e_{j} \end{array}\right]=\left[\begin{array}{cccccc} g_{\mathrm{ii}}H & g_{\mathrm{ij}}H & 0 & 0 & 0 & 0\\ g_{\mathrm{ji}}H & g_{\mathrm{jj}}H & 0 & 0 & 0 & 0\\ 0 & 0 & \sigma_{s_{i}}^{2}I_{s_{i}} & 0 & 0 & 0\\ 0 & 0 & 0 & \sigma_{s_{j}}^{2}I_{s_{j}} & 0 & 0\\ 0 & 0 & 0 & 0 & r_{\mathrm{ii}} & 0\\ 0 & 0 & 0 & 0 & 0 & r_{\mathrm{jj}} \end{array}\right]$$

where **g**_**ii**_, and **g**_**jj**_ were the additive‐genetic effects for the same trait in different parities *i* and *j*; **g**_**ij**_ and **g**_**ji**_ were additive genetic covariances between the same trait in different parities *i* and *j*; **H** was the combined (pedigree and genomics) relationship matrix as outlined above; $\sigma_{s_{i}}^{2}$ and $\sigma_{s_{j}}^{2}$ were the variances for the service sire effects on traits *i* and *j*, respectively, with the respective identity matrices $I_{s_{i}}$ and $I_{s_{j}}$; r_ii_ and r_jj_ were residual variances for the traits *i* and *j*, respectively.

The RRM in matrix notation was defined as follows:

$$y=\mathrm{Xb}+\mathrm{Zu}+\mathrm{Wp}+\left(\mathrm{SSs}\right)+e$$

where **y** was a vector for the same fertility trait in different lactations; **b** was a vector for fixed effects including herd‐year‐season of insemination and age of cow at insemination (in months), and fixed regressions on lactation number modelled with Legendre polynomials of order 2 representing the 3 coefficients intercept, first Legendre polynomial, second Legendre polynomial considering a standardisation between the minimal (parity 0 for heifers) and maximal parity 6; **u** was a vector for random regression coefficients for additive‐genetic effects modelled with Legendre polynomials of order 2 representing the 3 coefficients intercept, first Legendre polynomial, second Legendre polynomial considering a standardisation between the minimal (parity 0 for heifers) and maximal parity 6; **p** was a vector for random permanent environmental effects of the cow, **s** was a vector for random service sire effects for NRR56 DO; and **e** was a vector for random residual effects allowing heterogeneous residual variances in different parities. **X**, **Z**, **W**, and **SS** were incidence matrices for **b**, **u**, and **s**, respectively. Random effects were assumed to follow a normal distribution with zero means. The variance–covariance structure for random effects (CTFS without random service sire effects) was:

$$\mathrm{var}\left[\begin{array}{c} \begin{array}{c} u\\ p\\ s \end{array}\\ e \end{array}\right]=\left[\begin{array}{cccc} G⊗H & 0 & 0 & 0\\ 0 & \sigma_{p}^{2}I_{p} & 0 & 0\\ 0 & 0 & \sigma_{s}^{2}I_{s} & 0\\ 0 & 0 & 0 & \sigma_{e}^{2}I_{n} \end{array}\right]$$

where **G** was a 3 × 3 (co)variance matrix of random regression coefficients (intercept and the 2 coefficients for the Legendre polynomials) for the additive genetic effect; **H** was the combined relationship matrix as explained above; $\sigma_{p}^{2},$ $\sigma_{s}^{2}$ and $\sigma_{e}^{2}$ were variances for the permanent environmental, the service sire and the residual effect, respectively; **I**_**p**_, **I**_**s**_ and **I**_**n**_ were identity matrices for *p* cows, *s* service sires and *n* observations, respectively; and ⊗ denotes the Kronecker product.

For the estimation of genetic parameters and breeding values, GBLUP methodology as implemented in the BLUPF90 software packages (Aguilar et al. 2014), was applied.

With regard to model comparisons (i.e., RRM versus MTM), we correlated the respective parity‐specific estimated breeding values (EBV) considering the sires with at least 10 daughters (1875 sires for NRR56, 1849 sires for CTFS and 1801 sires for DO). From both modelling approaches, we created multiparity indexes for all three traits by combining the parity‐specific EBV with equal weights. Also the sire multiparity indexes were considered in the sire correlation analyses.

### 2.3 Correlations With Breeding Indexes From Official National Genetic Evaluations

Parity specific EBV (results from the RRM) for NRR56, CTFS and DO were standardised to relative breeding values (R_NRR56, R_CTFS and R_DO, respectively) using the mean and SD of the original breeding values from animals born in the year 2020. Relative breeding values for all three traits larger than 100 are favourable from a breeding perspective, i.e., increased non‐return rates, shorter days from calving to first service, and a shorter period for days open. The R_NRR56 of the 1875 sires, the R_CTFS of the 1849 sires and the R_DO of the 1801 sires with at least 10 daughters were correlated with their respective breeding values of the indexes included in the German total net merit index (RZG) from the official national genetic evaluation from 04/2022. The indexes (also standardised to a mean of 100 and a SD of 12 points) included: production (RZM), health (RZhealth), longevity (RZN), conformation (RZE), daughter fertility (RZR), calf fitness (RZcalfhealth) and calving traits for the paternal (RZKp) and for the maternal component (RZKm).

## 3 Results

### 3.1 Heritabilities and Variance Components for the Same Female Fertility Traits in Different Parities

**FIGURE 1:**
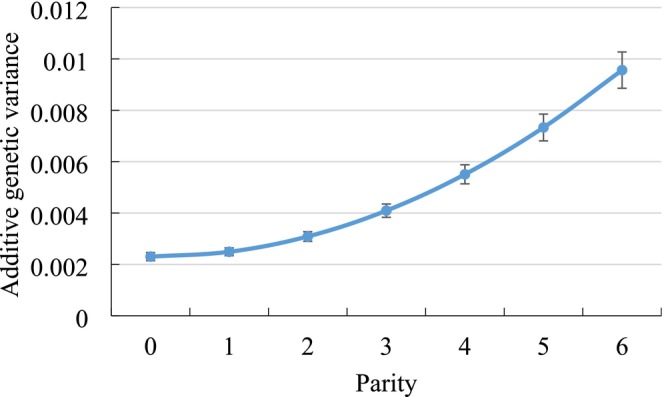
Additive‐genetic variances with respective SE for non‐return rate after 56 days (NRR56) in different parities (estimates from the random regression model). SE ranged from 0.00015 (parity 1) to 0.00071 (parity 6). [Colour figure can be viewed at wileyonlinelibrary.com]

**FIGURE 2:**
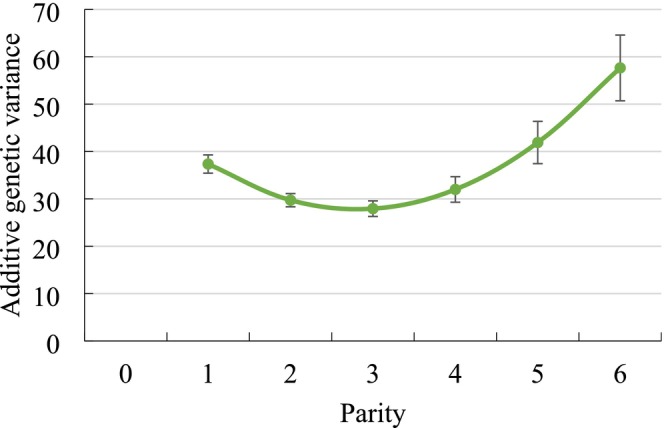
Additive‐genetic variances with respective SE for the interval from calving to first service (CTFS) in different parities (estimates from the random regression model). SE ranged from 1.38 (parity 2) to 6.93 (parity 6). [Colour figure can be viewed at wileyonlinelibrary.com]

**FIGURE 3:**
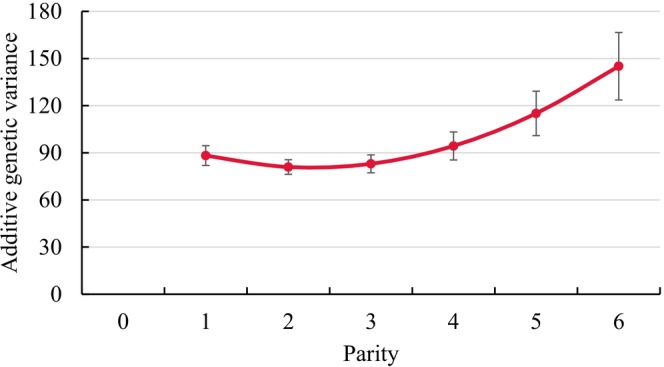
Additive‐genetic variances with respective SE for days open (DO) in different parities (estimates from the random regression model). SE ranged from 4.63 (parity 2) to 14.15 (parity 6). [Colour figure can be viewed at wileyonlinelibrary.com]

Genetic variances by parity with respective SE from the RRM application are displayed in Figure 1 for NRR56, in Figure 2 for CTFS and in Figure 3 for DO. In this regard, there was a general trend of increasing genetic variances with increasing lactation number, i.e., largest estimates in parities 5 and 6. Only for DO, the genetic variance was slightly larger in parity 1 than in the ongoing parities 2 and 3. Consequently, for NRR56 with additional consideration of heifer data, the genetic variance in heifers was substantially smaller than in all other parities of cows.

**FIGURE 4:**
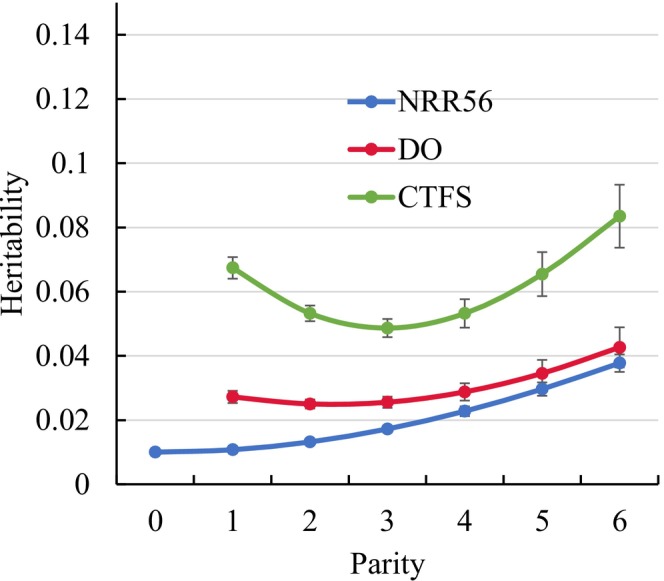
Heritabilities with respective SE for non‐return rate after 56 days (NRR56) (SE ranged from 0.001 to 0.003), for the interval from calving to first service (CTFS) (SE ranged from 0.002 to 0.006) and for days open (DO) (SE ranged from 0.001 to 0.006) in different parities (estimates from the random regression model). [Colour figure can be viewed at wileyonlinelibrary.com]

Increasing genetic female fertility variances with increasing lactation number explained the respective increases of heritabilities. The heritabilities for NRR56, CTFS and DO from the RRM are shown in Figure 4. Nevertheless, the heritabilities were quite small and in a narrow range from 0.01 (NRR56 in heifers) up to 0.08 (CTFS in parity 6). Smallest fluctuations in heritabilities by parity were observed for DO (0.027 in parity 1 up to 0.043 in parity 6).

The MTM estimates confirmed the general pattern for variance component estimates and heritabilities from the RRM, i.e., the trend of increasing genetic variances and heritabilities for NRR56 (Table 2), for CTFS (Table 3) and for DO (Table 4) with increasing lactation number. We also observed increasing service sire and residual variances for all 3 female fertility traits with increasing parity, but due to the stronger fluctuations of the additive‐genetic component, heritabilities were largest in late lactations. Nevertheless, the heritabilities were quite small and only differed marginally in different parities, i.e., in the range from 0.02 to 0.04 for NRR56, in the range from 0.04 to 0.06 for CTFS and in the range from 0.02 to 0.03 for DO.

**TABLE 2:** Variance components (service sire, additive‐genetic, residual) and heritabilities with corresponding SE for the non‐return rate after 56 days (NRR56) in different parities from the multiple‐trait model application.

Parity	Service sire	SE	Additive‐genetic	SE	Residual	SE	Heritability	SE
Heifers	0.001	0.001	0.005	0.001	0.210	0.004	0.023	0.001
1	0.002	0.001	0.007	0.001	0.230	0.003	0.028	0.001
2	0.001	0.001	0.007	0.001	0.236	0.005	0.030	0.001
3	0.002	0.001	0.008	0.001	0.234	0.005	0.033	0.001
4	0.001	0.000	0.009	0.002	0.234	0.005	0.037	0.001
5	0.003	0.001	0.010	0.001	0.234	0.005	0.039	0.001
6	0.006	0.002	0.010	0.001	0.234	0.005	0.041	0.001

**TABLE 3:** Variance components (service sire, additive‐genetic, residual) and heritabilities with corresponding SE for the interval from calving to first service (CTFS) in different parities from the multiple‐trait model application.

Parity	Additive‐genetic	SE	Residual	SE	Heritability	SE
1	18.57	5.87	390.74	9.25	0.045	0.015
2	14.54	2.66	367.79	4.98	0.038	0.013
3	18.93	2.61	407.69	5.64	0.044	0.014
4	22.24	4.18	410.27	5.47	0.051	0.017
5	26.89	3.57	420.64	9.43	0.060	0.018
6	27.36	5.01	422.28	11.03	0.061	0.018

**TABLE 4:** Variance components (service sire, additive‐genetic, residual) and heritabilities with corresponding SE for days open (DO) in different parities from the multiple‐trait model application.

Parity	Service sire	SE	Additive‐genetic	SE	Residual	SE	Heritability	SE
1	9.30	6.35	52.31	11.05	2310.8	45.17	0.022	0.016
2	16.39	8.63	55.85	9.95	2548.5	31.17	0.021	0.015
3	22.50	8.93	57.26	10.16	2598.1	39.58	0.021	0.016
4	19.27	7.60	62.09	6.99	2642.4	56.10	0.023	0.018
5	29.31	13.47	70.14	6.97	2744.2	56.68	0.025	0.018
6	30.60	21.43	92.37	20.71	2861.5	87.07	0.031	0.017

### 3.2 Genetic Correlations Between the Same Fertility Traits From Different Parities

**FIGURE 5:**
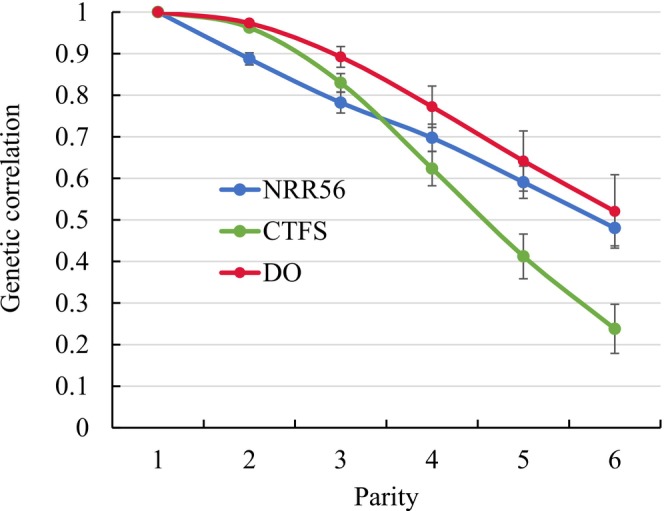
Genetic correlations (estimates from the random regression model) with respective SE between non‐return rate after 56 days (NRR56) in parity 1 with NRR56 in all other parities (SE ranged from 0.01 to 0.04), between the interval from calving to first service (CTFS) in parity 1 with CTFS in all other parities (SE ranged from 0.01 to 0.05), and between days open (DO) in parity 1 with DO in all other parities (SE ranged from 0.01 to 0.07). [Colour figure can be viewed at wileyonlinelibrary.com]

The RRM application enabled the estimation of genetic correlations between the same fertility traits from different parities, e.g., the genetic correlation between NRR56 in heifers with NRR56 in parity 1, between NRR56 in parity 1 with NRR56 in parity 2, etc. From the broad grid of genetic correlation combinations, we depicted the genetic correlations between the lowest cow parity number (parity 1) and all other parities for the same trait (see Figure 5). In this regard, the genetic correlations between the same traits from adjacent parities were close to 1 (0.89 between parity 1 NRR56 and parity 2 NRR56, 0.96 between parity 1 CTFS and parity 2 CTFS, and 0.97 between parity 1 DO and parity 2 DO), but gradually declined with increasing parity distances. Consequently, the smallest genetic correlations were estimated between the same trait from the earliest cow parity 1 and the latest parity 6. In this regard, the smallest genetic correlations were 0.48 (between parity 1 NRR56 and parity 6 NRR56), 0.24 (between parity 1 CTFS and parity 6 CTFS) and 0.52 (between parity 1 DO and parity 6 DO). Small genetic correlations were also identified between NRR56 in heifers and NRR56 in all cow parities, i.e., 0.40 with parity 1, 0.34 with parity 2, 0.31 with parity 3, 0.25 with parity 4, 0.23 with parity 5, and 0.20 with parity 6. The general genetic correlation pattern, i.e., quite large estimates for neighbouring parities, but a substantial decline for parities at greater distances, was also observed for other parity combinations and the same fertility traits.

The genetic correlations between same traits in different parities from the MTM are given in Table 5 for NRR56, in Table 6 for CTFS, and in Table 7 for DO. The genetic correlations generally support the estimate pattern from the RRM, i.e., a decline with increasing parity distances (apart from a few exceptions). However, the genetic correlations differences were smaller compared to the respective estimates from the RRM. The genetic correlations between NRR56 in heifers and NRR56 in all cow parities were in a narrow range from 0.26 (heifers and parity 3 cows) to 0.42 (heifers and parity 1 cows) (Table 5). Regarding NRR56, the genetic correlations among different cow parities were generally larger than 0.57, with a maximal value of 0.79 (parity 1 with parity 2).

**TABLE 5:** Genetic correlations with respective SE (below in brackets) between non‐return rate after 56 days (NRR56) from different parities from the multiple‐trait model application.

Parity	Parity						
	Heifers	1	2	3	4	5	6
Heifers		0.42 (0.056)	0.33 (0.057)	0.26 (0.066)	0.29 (0.052)	0.33 (0.044)	0.32 (0.047)
1			0.77 (0.053)	0.70 (0.064)	0.64 (0.062)	0.59 (0.059)	0.57 (0.064)
2				0.78 (0.054)	0.69 (0.063)	0.77 (0.065)	0.70 (0.070)
3					0.74 (0.064)	0.71 (0.067)	0.69 (0.053)
4						0.78 (0.063)	0.64 (0.067)
5							0.66 (0.057)

**TABLE 6:** Genetic correlations with respective SE (below in brackets) between the interval from calving to first service (CTFS) from different parities from the multiple‐trait model application.

Parity	Parity					
	1	2	3	4	5	6
1		0.77 (0.071)	0.70 (0.064)	0.64 (0.068)	0.59 (0.076)	0.57 (0.062)
2			0.78 (0.075)	0.69 (0.062)	0.77 (0.075)	0.70 (0.068)
3				0.75 (0.072)	0.74 (0.070)	0.69 (0.068)
4					0.78 (0.073)	0.64 (0.058)
5						0.66 (0.069)

**TABLE 7:** Genetic correlations with respective SE (in brackets below) between days open (DO) from different parities from the multiple‐trait model application.

Parity	Parity					
	1	2	3	4	5	6
1		0.63 (0.080)	0.62 (0.083)	0.64 (0.077)	0.55 (0.076)	0.63 (0.089)
2			0.73 (0.079)	0.71 (0.087)	0.65 (0.085)	0.59 (0.086)
3				0.60 (0.080)	0.61 (0.081)	0.67 (0.089)
4					0.51 (0.074)	0.50 (0.070)
5						0.75 (0.080)

Regarding the female fertility interval traits, the genetic correlations between the same trait in different parities were in a narrow range from 0.57 (parity 1 with parity 6) to 0.78 (parity 2 with parity 3) for CTFS (Table 6), and from 0.51 (parity 4 with parity 5) to 0.75 (parity 5 with parity 6) for DO (Table 7).

### 3.3 Correlations Between Breeding Values and Indexes From the Multiple Trait and the Random Regression Model

Table 8 displays the correlations between EBV for the same traits and parities from the RRM and MTM, considering the sires with at least 10 daughters. The agreement in parity‐specific EBV from both modelling approaches was quite large for the traits from the cow parities with correlation coefficients in the range from 0.93 (parity 1) to 0.87 (parity 6) for NRR56, in the range from 0.96 (parity 1) to 0.91 (parity 6) for CTFS, and in the range from 0.94 (parity 1) to 0.90 (parity 6) for DO. The smallest EBV correlation was found for NRR56 in heifers with a coefficient of 0.85. Accordingly, the correlations between the multiparity indexes for all three traits were large with 0.91 for NRR56 and with 0.92 for CTFS and DO (Table 8).

**TABLE 8:** Breeding value correlations between the parity‐specific breeding values and correlations between multiparity indexes for the non‐return rate after 56 days (NRR56), for the interval from calving to first service (CTFS) and for days open from the multiple‐trait model (MTM) and the random regression model (RRM) considering the sires with at least 10 daughters.

Trait	Heifer	Parity						Multiparity index
		1	2	3	4	5	6	
NRR56	0.85	0.93	0.93	0.90	0.89	0.90	0.87	0.91
CTFS		0.96	0.94	0.92	0.93	0.92	0.91	0.92
DO		0.94	0.93	0.91	0.90	0.91	0.89	0.92

### 3.4 Correlations Between Breeding Values for Female Fertility Traits From Different Parities With Breeding Indexes

Sire breeding value correlations between R_NRR56 with the official breeding indexes (Table 9), R_CTFS with the official breeding indexes (Table 10) and R_DO with the official breeding indexes (Table 11), were in a narrow range for the same female fertility traits in different cow parities. Largest differences were found for NRR56 when comparing the breeding value correlations in heifers with breeding correlations from the cow parities (Table 9). For example, the correlation between R_NRR56 in heifers with RZG was 0.05, but in the range from 0.18 to 0.46 between R_NRR56 from the different cow parities with RZG. A general antagonistic relationship was found between RZM with R_NRR56 in heifers (−0.08) and with R_NRR56 in all parities (−0.09 to −0.25). The largest favourable breeding value correlations were identified between R_NRR56 from all parities with RZR in the range from 0.35 (heifers) to 0.75 (parity 3). Moderate and favourable were the breeding value correlations between R_NRR56 with longevity (RZN) and with the overall health index (RZHealth), again with largest differences between associations in heifers and in cow parities.

**TABLE 9:** Breeding values correlations between the relative breeding value for non‐return rate after 56 days (NRR56) in different parities (estimates from the random regression model) and breeding indexes of 1875 sires with at least 10 daughters.

Index^a^	Heifer	Relative breeding values for NNRR56 in different parities					
		1	2	3	4	5	6
RZG	0.05	0.18	0.28	0.25	0.38	0.36	0.46
RZM	−0.08	−0.12	−0.09	−0.15	−0.12	−0.25	−0.22
RZN	0.09	0.31	0.24	0.25	0.38	0.43	0.46
RZE	−0.01	−0.11	−0.08	−0.14	−0.16	−0.22	−0.17
RZR	0.35	0.59	0.62	0.65	0.61	0.75	0.70
RZHealth	0.10	0.23	0.20	0.31	0.33	0.37	0.39
RZcalfhealth	−0.01	0.06	0.03	0.10	0.11	0.13	0.15
RZKp	0.01	0.12	0.17	0.13	0.19	0.24	0.22
RZKm	0.04	0.16	0.15	0.23	0.24	0.26	0.24

^a^ RZcalfhealth = calf fitness index, RZE = conformation index, RZG = overall net merit index, RZhealth = health index, RZKm = index for calving ease maternal, RZKp = index for calving ease paternal, RZM = production index, RZN = longevity index, RZR = daughter fertility index.

**TABLE 10:** Breeding values correlations between the relative breeding value for the interval from calving to first service (CTFS) in different parities (estimates from the random regression model) and breeding indexes of 1849 sires with at least 10 daughters.

Index^a^	Relative breeding values for CTFS in different parities					
	1	2	3	4	5	6
RZG	0.16	0.11	0.11	0.09	0.06	0.07
RZM	−0.40	−0.34	−0.30	−0.31	−0.26	−0.22
RZN	0.28	0.33	0.37	0.40	0.41	0.47
RZE	0.00	−0.03	−0.05	−0.07	−0.04	−0.03
RZR	0.45	0.46	0.49	0.48	0.52	0.59
RZHealth	0.25	0.23	0.38	0.34	0.40	0.47
RZcalfhealth	0.03	−0.01	−0.05	0.00	−0.02	0.00
RZKp	0.07	0.04	0.08	0.03	0.01	0.03
RZKm	0.11	0.10	0.05	0.07	0.03	0.01

^a^ RZcalfhealth = calf fitness index, RZE = conformation index, RZG = overall net merit index, RZhealth = health index, RZKm = index for calving ease maternal, RZKp = index for calving ease paternal, RZM = production index, RZN = longevity index, RZR = daughter fertility index.

**TABLE 11:** Breeding values correlations between the relative breeding value for days open (DO) in different parities (estimates from the random regression model) and breeding indexes of 1801 sires with at least 10 daughters.

Index^a^	Relative breeding values DO in different parities					
	1	2	3	4	5	6
RZG	0.18	0.23	0.20	0.17	0.17	0.15
RZM	−0.27	−0.22	−0.16	−0.15	−0.15	−0.14
RZN	0.39	0.41	0.56	0.54	0.50	0.50
RZE	−0.16	−0.10	−0.11	−0.09	−0.13	−0.09
RZR	0.61	0.74	0.68	0.77	0.87	0.80
RZHealth	0.32	0.26	0.40	0.38	0.37	0.46
RZcalfhealth	0.07	0.03	0.01	0.03	0.00	0.01
RZKp	0.17	0.20	0.26	0.23	0.28	0.27
RZKm	0.26	0.25	0.20	0.20	0.17	0.18

^a^ RZcalfhealth = calf fitness index, RZE = conformation index, RZG = overall net merit index, RZhealth = health index, RZKm = index for calving ease maternal, RZKp = index for calving ease paternal, RZM = production index, RZN = longevity index, RZR = daughter fertility index.

With regard to R_CTFS breeding value correlations with all other indexes (Table 10), only minor to moderate differences were observed across parities. The antagonistic associations between R_CTFS and RZM declined from parity 1 (−0.40) to parity 6 (−0.22). In analogy with non‐return rates, the strongest favourable associations were found between R_CTFS and RZN in the range from 0.25 to 0.47, with RZHealth in the range from 0.23 to 0.47, and with RZR in the range from 0.45 to 0.59. Due to the favourable effect of R_CTFS on other functional traits or indexes in all parities, but the consistently unfavourable associations with RZM, the breeding value correlations with RZG were close to zero.

Breeding value correlations between R_DO with all other indexes (Table 11) reflect the pattern as presented for R_CTFS, displaying only minor to moderate deviations in correlation coefficients for the same indexes with R_DO in different cow parities. Quite strong and favourable were the correlations between R_DO in different parities with RZR in the range from 0.61 to 0.87. In analog with CTFS, moderate and favourable correlations were found between R_DO with RZN in the range from 0.39 (in parity 1) to 0.56 (in parity 3), and between R_DO with RZHealth in the range from 0.26 (in parity 2) to 0.46 (in parity 6). Antagonistic in all parities were the breeding value correlations between R_DO with RZM in the range from −0.27 (in parity 1) to −0.14 (in parity 6).

## 4 Discussion

### 4.1 Heritabilities and Variance Components for the Same Female Fertility Traits in Different Parities

From both modelling approaches (RRM and MTM applications), we found slight increases in additive‐genetic variances and heritabilities in all three female fertility traits with increasing parities, with the smallest estimates for NRR56 in heifers. Liu et al. (2008) applied MTM for NRR56 and distinguished between heifers and cows. In their study, the NRR56 heritability was only slightly larger in cows (0.015) than in heifers (0.012). For the cow female fertility interval traits, the heritabilities reported by Liu et al. (2008) reflect the estimates from our present study. Slight deviations in heritabilities from different studies might be due to the statistical modelling approach, e.g., treating the service sire as fixed (Liu et al. 2008) or as a random effect as done in our study, or due to the modelling of genetic relationships (pedigree based versus genomics) (Shabalina et al. 2020). Recently, also Zhu et al. (2023) indicated smaller variance components and heritabilities for female fertility traits in heifers than in cows, but on a generally very low level, e.g., 0.0014 for NRR56 in heifers and 0.002 for NRR56 in cows. Muuttoranta et al. (2019) applied a multiple‐trait multiple‐lactation model for female fertility traits in Nordic Holstein cows, and reported the smallest heritabilities in heifers and increasing heritabilities with increasing lactation number, also for the female fertility interval traits as considered in our present study. Interestingly, such effects were negligible in Nordic Red Dairy cattle, indicating breed‐specific effects on female fertility variance components in different parities. Albeit the only small differences between NRR56 heritabilities observed in heifers and cows, the slight increase in genetic variations with increasing parity seems to be a bit surprising when addressing aspects of natural and artificial selection. Usually, as indicated by König et al. (2005) for claw disorders, diseased cows have a greater risk for disposals before reaching the subsequent parity, narrowing genetic trait variation. The same might be the case for female fertility, being a major reason for involuntary cow cullings (Shabalina et al. 2019). Doublet et al. (2019) outlined the effects of artificial selection on declining genetic diversity measurements including genetic variations. Furthermore, with regard to female fertility variations, natural selection is a major driving component, especially in harsh environments, supporting Darwin's concept of ‘survival of the fittest’, with effects on reproduction rates with progressing time in specific genetic lines (Paul 1988). However, for studying the effects of selection, genetic variations in heifer fertility over years, or in cow fertility in specific parities over years, might be a better indicator than comparing the same traits in cows and in heifers. Differing genetic parameters in the same trait in heifers and cows might indicate a differing genetic trait architecture and differing gene activities for fertility with aging, as outlined via gene expressions in humans (Zhang et al. 2020).

A main focus was the comparison of MTM estimates with RRM estimates along the parity or aging trajectory. In this regard, Paneru et al. (2024) recommended RRM applications for weight traits in sheep, justified by a more accurate capturing of the genetic (co)variance structure and more accurate genetic evaluations over time than MTM. However, for weight traits in sheep, the repeated measurements were in closer intervals compared to the different parities in the present female fertility study, and the number of repeated weight measurements per sheep (minimum: 4 records per animal) was generally larger than the repeated female fertility structure in cows (maximum: 6 records per animal). Interestingly, also in the sheep study by Paneru et al. (2024) and in analogy with NRR56, CTFS and DO in the present study, additive‐genetic variances and heritabilities from the RRM increased with increasing age (apart from the very early beginning). However, increased genetic parameters at the ‘extreme ends’ of the age scale could be due to limited data to model the covariance function (Meyer 2004). In the present study, the number of observations and animals was quite large in all parities. Furthermore, in the present study, the genetic parameter pattern by parity as obtained from the RRM is confirmed through the estimates from the MTM. Both applications RRM and MTM enabled the estimation of breeding values of animals without records in distinct parities or age classes. In this regard, with a focus on an environmental climate scale, Bohlouli et al. (2018) indicated more accurate predictions in case of missing records when applying genomic RRM compared to other modelling approaches. Paneru et al. (2024) indicated very large breeding value correlations for weight traits from the RRM and the MTM at the same age classes. Furthermore, Paneru et al. (2024) showed very similar genetic parameter estimates for the same ages based on RRM and MTM applications, supporting the similarities in RRM–MTM comparisons for variance components and heritabilities for the female fertility traits in our study. Nevertheless, the great advantage when applying an RRM compared to an MTM is the ability of genetic predictions and genetic parameter estimations at any point of the continuous time scale or along environmental scales. This is especially the well‐known case for dense longitudinal data structures. Such concepts were carefully evaluated by Yin, Pimentel, et al. (2014), displaying the advantages of RRM also for only a few (maximum 5) records per cow for consecutive climatic levels.

### 4.2 Genetic Correlations Between the Same Fertility Traits From Different Parities

The decline of genetic correlations in the same fertility traits with increasing parity distance is well‐known for other cow traits and data structures, e.g., for monthly test‐day production records (e.g., Swalve 1995, 2000) or for cattle weights along the age scale (Yin and König 2017), but also for production and health indicator traits along a climatic trajectory (Bohlouli et al. 2018). The genetic correlations from the RRM are confirmed through the respective estimates from the MTM as additionally applied in the present study, and through previous MTM publications. For example, Liu et al. (2017) indicated the effect of proximity in lactation numbers on the shared genetic mechanisms among traits. However, in contrast, Liu et al. (2017) estimated negative genetic correlations between non‐return rates in heifers and in cows. The only moderate genetic correlations between the same female fertility traits from different cow parities (minimal coefficient: 0.24 for CTFS in parity 1 with CTFS in parity 6 from the RRM) suggest indicating specific breeding values for specific parities, which will contribute to more precise mating and selection strategies.

With regard to NRR56 and RRM applications, our estimates are in agreement with results by Averill et al. (2006), but they based their study on a denser data structure within lactations for the success of an insemination. In contrast, Averill et al. (2006) applied threshold methodology for binary female fertility traits, but for binary NRR56, we used a linear RRM. With regard to heritabilities, differences on the underlying liability scale and on the observed scale are expected (Dempster and Lerner 1950). However, in the case of NRR56 with intermediate frequencies for ‘pregnant’ or ‘non‐pregnant’, and for such a large dataset, only minor differences from linear and threshold model applications are expected. Especially with regard to genetic correlations, estimates from linear and threshold models are theoretically expected to be the same, as shown by Vinson and Kliewer (1976) for type traits. From a random regression modelling perspective, further differences in (co)variance components might be due to the applied covariance function and the polynomial order for the time dependent variable (e.g., Meyer 2005). In the present study, we ran RRM with Legendre polynomials of order 2, which probably explains increasing variances at the extreme ends of the time scale and smaller genetic correlations between the same traits from different parities. However, such a modelling approach can be justified with the large dataset in the present study, because a strong impact of the polynomial order on genetic parameter estimates was only observed for small datasets and small herd sizes with small contemporary groups (Yin, Bapst, et al. 2014). Furthermore, the chosen covariance function or polynomial structure should be interpreted in the context of computing time. All estimations and related computations were conducted on high‐performance computing systems with 192 GB RAM and 24‐core CPU nodes. In preliminary runs using first order Legendre polynomials, evaluations took 7–8 days per trait, but 10–12 days per trait for the second order Legendre polynomials. Regarding computation time, the MTM did not perform better than the RRM.

The correlations between the parity‐specific sire EBV from the MTM with the respective sire EBV from the RRM were throughout larger than 0.87 for the cow fertility traits in the present study. The EBV correlation coefficient was slightly smaller in heifers with 0.84 for NRR56. In consequence, the large EBV correlations imply only minor re‐rankings of sires due to a change from an MTM approach towards an RRM. Interestingly, regarding the cow parities, the smallest correlations were observed in parity 6, but still displayed coefficients larger than 0.87. An explanation of larger EBV deviations in late parities from both modelling approaches might address the data structure due to the effect of selection. Only the high fertile cows are kept in the herds in late lactations. In this regard, the possible effect of pre‐selection on biased EBV was intensively discussed in horse breeding for single‐trait models (e.g., Bugislaus et al. 2006). In such context, an RRM with greater flexibility for the definition of covariance functions might be superior over MTM.

### 4.3 Breeding Value Correlations Between Female Fertility Traits From Different Parities With Breeding Indexes

With regard to EBV correlations between R_NRR56, R_CTFS and R_DO with the indexes from the German official national genetic evaluation, minor to moderate differences were identified across the cow parities. Stronger differences in correlation coefficients were observed when comparing heifers with cows. In consequence, a general separation between female fertility heifer traits and female fertility cow traits as being current practice in most of the national genetic evaluations worldwide, is justified (e.g., Jorjani 2006; Gredler et al. 2007).

From a physiological perspective, it is interesting to note that antagonistic relationships between productivity (in terms of RZM) and female fertility traits do exist over the whole cow lifespan, i.e., in heifers up to parity 6. These unfavourable breeding value correlations based on the RRM approach confirm estimates from previous studies with a focus on single specific lactations (Jayawardana et al. 2022), on repeatability models (Chafai et al. 2024) or on MTM (Sewalem et al. 2010). In Jersey crosses kept in stressful tropical conditions, the genetic correlations between 305‐days milk yield with numbers of services per conception, days open and calving interval were large and positive (Roy et al. 2024), supporting the antagonistic relationships between productivity and female fertility in breeds other than Holstein Friesian and in differing production systems.

Consistent favourable correlations between breeding values for female fertility traits and longevity (RZN) were identified across all parities. Accordingly, moderately favourable genetic correlations between longevity (in terms of the length of productive life) and female fertility traits (DO, CTFS and the interval from first to last insemination) were reported by Zavadilová and Zink (2013) in Czech Holsteins by applying bivariate linear animal models. Hence, the estimates from our applied RRM reflect the correlation pattern between female fertility and longevity traits as outlined before on the basis of more simple modelling approaches. Also, breeding value correlations were consistent and favourable with the overall health index (RZHealth) across all parities, but a weak correlation (0.10) was found between NRR56 in heifers and RZHealth. The overall health index (RZHealth) includes cow health traits that are directly related to female fertility, i.e., endometritis or retained placenta, explaining the favourable breeding value correlations in all cow parities. In consequence, Gernand and König (2017) estimated quite strong genetic correlations between fertility diseases and the female fertility traits CTFS, the interval from first service to pregnancy and the interval from calving to pregnancy. Respective genomic associations between fertility and uterine diseases were inferred by May et al. (2021).

The quite strong EBV correlations between female fertility traits from different parities with the overall fertility index (RZR) are the logical consequence, because RZR is strongly determined through the single female fertility composites (Pasman et al. 2006).

### 4.4 Further Explanations for Differing Genetic Parameters in Different Parities

In quantitative‐genetic studies, the most common argument for differing genetic parameters with progressing time is that different genes might be ‘switched on’ or ‘switched off’. For fertility traits in cattle, such explanations were confirmed via differing gene expression patterns (e.g., Cai et al. 2019). Recently, alterations of genetic parameters and variance components with progressing time were discussed in the context of epigenomic mechanisms, induced by environmental stressors during early pregnancy (Kipp, Brügemann, Yin, et al. 2021). Such postulations are mostly based on observations made in humans, e.g., the effect of prenatal famine in the embryonic stage on epigenetic modifications, and in causality, on chronic diseases related to infertility (Heijmans et al. 2008). Kipp, Brügemann, Yin, et al. (2021) studied heat stressors during pregnancy, and identified time‐lagged genotype × heat stress interactions and alterations of (co)variance components especially for NRR56. Interestingly, in another study by Kipp, Brügemann, Zieger, et al. (2021), the long‐lasting effects were strongest for traits recorded in late parities (e.g., longevity). In contrast, Halli et al. (2021) reported only minor effects on early available weight traits in beef cattle. Physiological explanations in both studies (Kipp, Brügemann, Yin, et al. 2021; Halli et al. 2021) addressed heat stressors during maturation and late pregnancy and their associations with genome methylation patterns, with the strongest effects on pregnant cows in their first parity. An argument in this regard might be an additional stress component due to an increased energy deficit (Gross and Bruckmeier 2019). First parity cows need energy for milk production and for growth, causing stronger clinical signs of metabolic stress and metabolic disorders compared to adult cows. Accordingly, López‐Catalina et al. (2024) hypothesised that ‘calves gestated by nonlactating mothers have a different methylation profile than those gestated by lactating cows’. Such epigenomic aspects as outlined above are speculative, but moderate to strong genetic parameter alterations for female fertility traits across parities and related physiological explanations in other species, suggest ongoing genomic research in this regard.

## 5 Conclusion

The application of RRM for NRR56, CTFS and DO along the time‐dependent parity scale resulted in reliable genetic parameters, which were confirmed via MTM. Especially for NRR56, heritabilities and variance components differed with regard to heifers and cow parities, supporting the approach to distinguish between heifer and cow female fertility in genetic evaluations. For all three female fertility traits (from the RRM as well as from the MTM), heritabilities and additive‐genetic variances increased with increasing parity. Genetic correlations between the same fertility traits from different parities declined with increasing parity distance, also across the cow parity scale. The differing genetic background of fertility traits in different parities resulted in differing breeding value correlations between R_NRR56, R_CTFS and R_DO and other breeding indexes along the parity trajectory. However, larger differences in this regard were only found for NRR56 in heifers and in cows. Correlations between cow parity‐specific EBV for the same trait from both modelling approaches MTM and RRM were larger than 0.87.

## Conflicts of Interest

The authors declare no conflicts of interest.

## Data Availability

The data that support the findings of this study are available on request from the corresponding author.
